# Narrative mobile video game-based cognitive training to enhance frontal function in patients with mild cognitive impairment

**DOI:** 10.1038/s41598-024-84086-9

**Published:** 2025-01-02

**Authors:** Yeseul Choi, Jae-Sung Lim, Hagyun Choi, Yong Hoe Ryu, Eunkyung Seong, Inseok Park, Dong Won Kang, Jae-Hong Lee, Dong-Wha Kang

**Affiliations:** 1 Nunaps Inc., Seoul, Korea; 2 Department of Neurology, Asan Medical Center, University of Ulsan College of Medicine, Seoul, Korea

**Keywords:** Neuroscience, Cognitive ageing, Cognitive neuroscience

## Abstract

Although cognitive training has been proposed as a possible therapeutic modality for mild cognitive impairment (MCI), most serious games focus on specific tasks. This study aimed to investigate the feasibility and efficacy of narrative video game-based cognitive intervention for MCI. A four-week (± 1-week) mobile game intervention was given to 17 MCI participants (mean age (SD) = 72.8(4.75)). At baseline and post-intervention, the participants received neuropsychological tests and a depression scale. Frontal function was assessed using the Corsi block-tapping test, Color Word Stroop Test, Controlled Oral Word Association Test, Digit Symbol Coding, and Trail Making Test-Elderly’s Version; depression was assessed using the Geriatric Depression Scale. User’s compliance and gaming experience were also evaluated. MCI patients showed significant improvements in frontal function, particularly in Digit Symbol Coding (mean ± SD, 0.47 ± 0.49, *p* = 0.007) and phonemic fluency (mean ± SD, 0.39 ± 0.55, *p* = 0.024). Each frontal subtest’s mean z-score was increased (mean ± SD, 0.44 ± 0.38, *p* = 0.008). Block span and depression scale remained unchanged. High adherence rates (122.35%) and favorable feedback on the gaming experience indicated that the game intervention’s usability boosted patients’ motivation and engagement. Our findings demonstrate that narrative game-based cognitive intervention was not only beneficial but also enjoyable for elderly MCI.

## Introduction

Although mild cognitive impairment (MCI) is acknowledged as an important pathological condition that leads to dementia including Alzheimer’s disease (AD)^1,2^, there is currently no pharmacological or non-pharmacological treatment that has been shown to be effective. As a result, proper and targeted management is urgently needed, especially given the rapidly increasing number of people with MCI and dementia^3^. The American Academy of Neurology’s updated practice guideline for MCI^4^stated that there is no standard treatment for the condition; instead, regular exercise or cognitive training is advised. This is because pharmacological treatments, such as cholinesterase inhibitors, have limited efficacy and risks of adverse events^5^.

There has been increasing interest in the effects of serious games (SGs) based on various neuropsychological tests on patients with AD and MCI. Previous findings indicated that SGs were beneficial for cognitive impairment, particularly in the areas of frontal and executive function^6,7^. There is also compelling evidence to suggest that SGs, when combined with a minigame-style task set, enhance cognitive performance on trained tasks^8^. It is, however, difficult to sustain user motivation over time, because most SGs are task-oriented. Furthermore, given that most MCI and AD patients are elderly, there may always be a concern that these individuals have psychological barriers to playing games, struggle to follow rules, and eventually lose interest in them.

Early impairment in frontal function is considered a sensitive predictor of cognitive deterioration in MCI^9–11^. According to prior studies, frontal function is closely related to the ability to perform activities of daily living (ADLs) and maintain independence^12^. Since ADL performance is a strong indicator distinguishing between MCI and dementia, training frontal function would be effective in both preventing dementia and improving cognitive function in MCI patients. In addition, 36–43% of individuals with MCI show depressive symptoms according to previous studies^13^, and monitoring mood changes during the intervention might be important because the narrative and background music of the game could potentially influence the emotional state of the participants^14^. Depressive symptoms can potentially limit a patient’s ability to fully participate in treatment^15^. Thus, we consider it important to determine whether depressive symptoms affected during the intervention period and to assess whether the emotional content of the game had any negative impact on participants’ mood.

With these considerations in mind, we hypothesized that an interactive adventure video game, with a narrative designed to immerse the patient and allow them to take on the role of the protagonist solving mysteries, could motivate MCI patients to continue engaging with cognitively demanding tasks. Based on this hypothesis, this exploratory clinical trial aimed to investigate whether a narrative-driven interactive adventure video game could improve treatment adherence and frontal function in MCI participants.

## Results

### Efficacy of the intervention

**Fig. 1:**
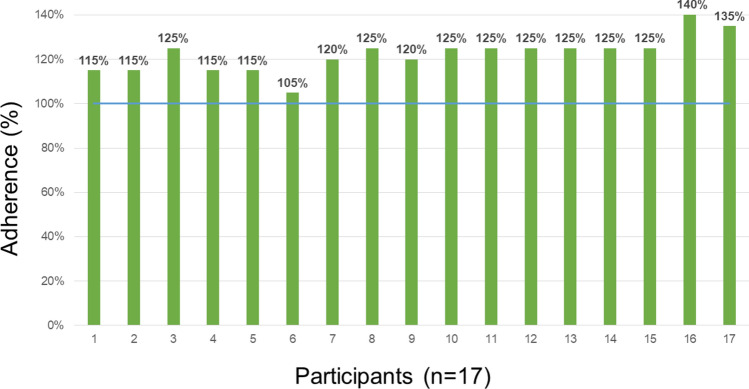
An overview of the adherence of all participants. When participants completed the intervention five times per week for 4 weeks (± 1 week), the compliance rate was recorded as 100%. The possible highest rate was 140%, if the participant played seven times per week.

The demographic and clinical factors of the patients are summarized in Table 1**.** All participants completed the cognitive intervention sessions for 4 weeks (± 1 week). Our intervention should be performed at least five times per week, but it is also permitted to be conducted up to seven times a week, which is considered the highest compliance rate (140%). Figure 1 shows an overview of the adherence of the participants in the study. The average completion rate for the intervention was 122.35% (Fig. 1).

**Table 1:** The demographic and clinical factors of the participants.

	**Participants** **(n = 17)**
Age (years)	73.1 $\pm$ 5.0
Education (years)	11.1 $\pm$ 3.8
Female	9 (52.9%)
Global Deterioration Scale	2.7 $\pm$ 0.6
Follow up (days)	35.6 $\pm$ 5.6

Numbers denote mean ± standard deviations or frequencies (proportions) as appropriate.

As shown in Table 2, there were significant improvements in Controlled Oral Word Association Test (COWAT) Phonemic fluency, Digit Symbol Coding (DSC), and Trail Making Test-Elderly’s Version (TMT-E) Part B. The most significant increase was seen in DSC performance (P = 0.009) followed by COWAT phonemic fluency (P = 0.003), and TMT-E Part B (P = 0.01). After applying multiple comparison correction, the COWAT phonemic fluency and DSC scores still showed significant changes (corrected P = 0.024 and 0.007, respectively; Table 2).

**Table 2:** Mean changes in efficacy measures from baseline to post-intervention within participants.

	**Baseline scores (n = 17)**	**Post-intervention scores (n = 17)**	**Change from baseline to post-intervention (n = 17)**	**P-value**	**Corrected P-value**
Frontal/Executive Function Subtests (Z-score)					
CWST **(**Color Reading)	0.12 $\pm$ 1.02	0.36 $\pm$ 0.65	0.25 $\pm$ 0.90	0.170	0.999
COWAT (Phonemic fluency)	−0.67 $\pm$ 0.69	−0.28 $\pm$ 0.76	0.39 $\pm$ 0.55	0.003	0.024
DSC	0.42 $\pm$ 0.97	0.90 $\pm$ 1.18	0.47 $\pm$ 0.49	0.001	0.007
TMT-E Part A Part B	0.08 $\pm$ 0.52 −0.39 $\pm$ 1.32	0.32 $\pm$ 0.65 0.46 $\pm$ 0.51	0.24 $\pm$ 0.69 0.85 $\pm$ 1.26	0.120 0.010	0.960 0.080
Mean z-score of frontal function tests	−0.09 $\pm$ 0.60	0.35 $\pm$ 0.53	0.44 $\pm$ 0.38	0.001	0.008
Corsi block-tapping test					
Block span	4.29 $\pm$ 0.69	4.41 $\pm$ 1.06	0.12 $\pm$ 1.05	0.675	0.999
GDS					
Total score	11.82 $\pm$ 4.64	9.06 $\pm$ 4.62	−2.76 $\pm$ 5.94	0.110	0.880

Scores of Frontal/Executive function subtests were investigated as z- scores. Both the Corsi block-tapping test and Geriatric Depression Scales were evaluated as raw scores, block span, and total scores, respectively. Numbers denote mean ± standard deviations.

CWST, color word Stroop test; COWAT, controlled oral word association test; DSC, digit symbol coding; TMT-E, trail-making test-elderly’s version; GDS, geriatric depression scale.

Overall, the mean z-score of all frontal function tests significantly increased from − 0.09 to 0.35 (mean (SD) = 0.44(0.38)) after training (uncorrected P = 0.001, corrected P = 0.008). About 90% of the patients (n = 15) showed an improvement in the mean z-score for frontal/executive function tests after training, and of those, eight patients reported a mean z-score change (mean (SD) = 0.76(0.25)), which was substantially higher than the average.

However, no significant difference was observed in Corsi block score between baseline and after the training. There were no patients with significant depression with a geriatric depression scale (GDS) score of 18 or higher at baseline, and the GDS score also did not significantly change after the intervention.

### User experience of the intervention

**Fig. 2:**
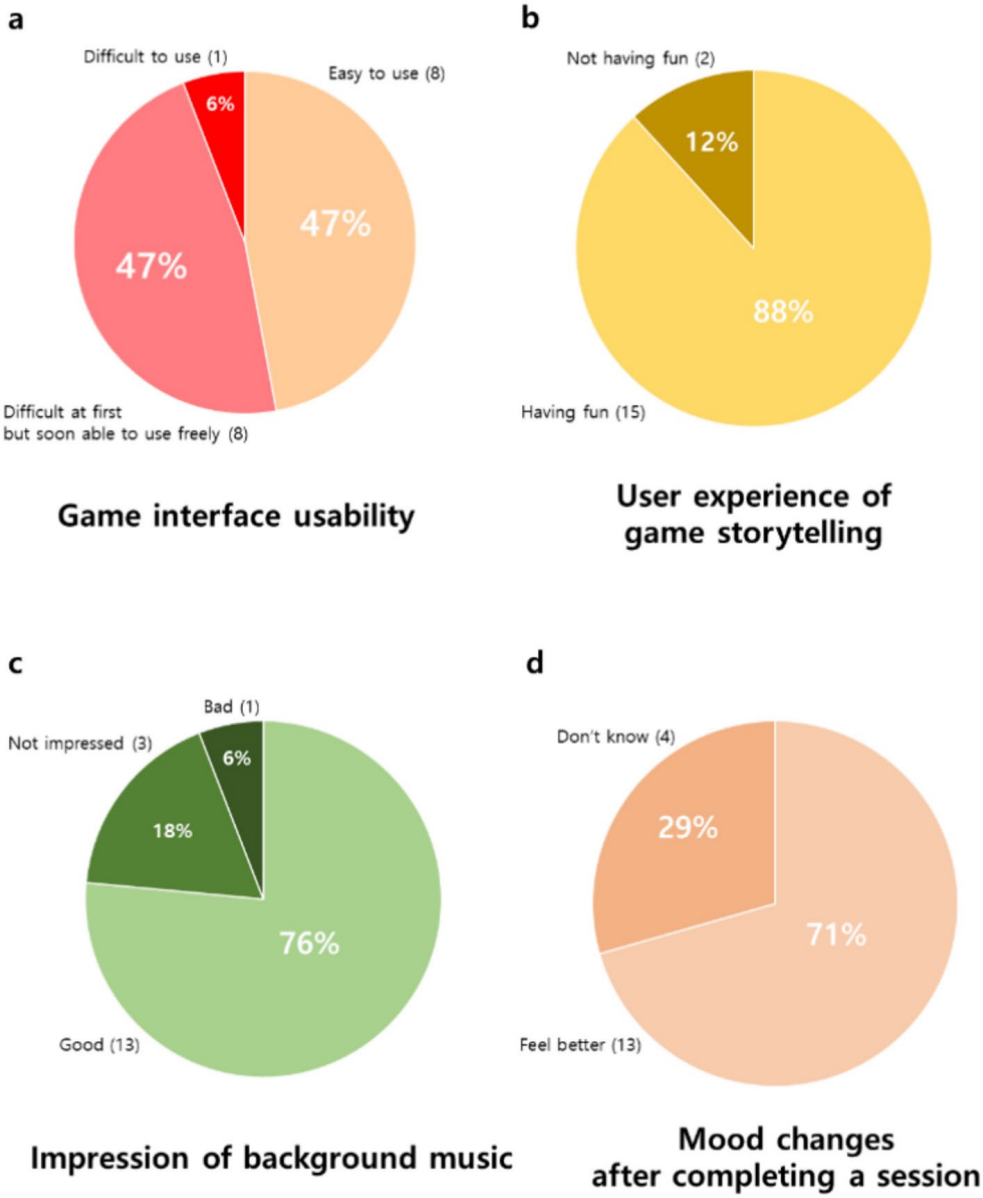
(**a**) Game interface usability of the intervention. Participants evaluated whether the game interface was easy to use or not. (**b**) User experience of game storytelling of the intervention. (**c**) Impression of the background music of the intervention. (**d**) Mood changes after completing a session of the intervention.

User experience interview results of the participants are shown in Fig. 2. Figure 2a shows that most participants responded they did not have serious trouble with the game interface, and all subjects used it without difficulty during the intervention. Regarding game storytelling, an important part of our intervention, Fig. 2b revealed that 88% of the participants stated that they enjoyed the game story. In addition, the background music, which is also part of the intervention, was considered good by approximately three-quarters of all participants (Fig. 2c**)**. Figure 2d shows that over 70% of participants experienced positive mood changes after completing one session per day. Most participants expressed positive feelings after the training such as willing to play after commercialization, willing to recommend this game to other MCI patients, or attaining self-efficacy (Table 3).

**Table 3:** Feedback for the game-based intervention from patients and their primary caregivers. All participants’ feedback was recorded but primary caregivers’ feedback was investigated only when they were accompanied by patients.

	**Yes**	**Yes** **(% of definite responses)**
Feedback from patients (n = 17)		
Willing to play after commercialization	16	94
Willing to recommend to other MCI patients	15	88
Attaining self-efficacy	15	88
Having fun	12	71
Having a motivation of subsequent study	10	59
Feedback from primary caregivers (n = 17)		
Hoping patients keep playing	8	47
Noticeable cognitive improvement in patients	6	35
Positive change in mood of patients	3	18
Willing to do by their self	3	18

We also obtained mostly positive feedback by interviewing the primary caregivers. The primary caregivers monitored the patients’ training during the sessions and reported on the patients’ level of engagement after the intervention by interviewing. Approximately half of the primary caregivers hoped the patients would keep playing this game-based intervention. Six primary caregivers noticed a cognitive improvement in their patients, and three individuals felt positive changes in the patients’ moods. Some primary caregivers commented that they wanted to participate in the intervention themselves as it seemed to be effective and interesting to play.

## Discussion

In this study, we observed that the narrative video game-based cognitive intervention greatly improved frontal function and showed high engagement in MCI individuals. Our findings showed that COWAT phonemic fluency and DSC were significantly improved, and that the mean z-score obtained by averaging the five subtests was likewise significantly higher. Furthermore, participants reported positive feedback regarding their gaming experience and stated that the game intervention was easy to use. These results suggest that video game-based training may enable elderly MCI subjects to interact at a high level.

Above all, the remarkable compliance rate in our study may be explained by the immersive video game-based nature of our cognitive training and the unmet need for appropriate cognitive training intervention in current clinical practice. Elderly participants with MCI stated that, despite their lack of gaming experience, they were able to easily get used to the game interface and that, with the narrative providing a framework for the training, they were completely absorbed in the intervention, which improved their ability to concentrate and train their frontal/executive functions in more realistic activities^16^. Most participants and their primary caregivers expressed a desire to use the treatment continually to treat cognitive impairment and, if possible, to promote it to others. It is common knowledge that video games offer a more complex, compelling, and alluring narrative than other forms of media^17^. It is also clear that seniors are not an exception, and this research suggests that senior gaming can have positive and productive effects on mental health.

The most clinically significant outcome might be the improvement in COWAT phonemic fluency and DSC. These two subtests are sensitive for the detection of psychomotor speed decline, which is a frontal dysfunction that may manifest in the early stage of MCI^18^. Particularly, DSC has been proposed as an important indicator of alterations in symptoms associated with cognitive impairment and even of daily functions^19^. When combined, the gains in phonemic fluency and DSC suggest that MCI patients have improved frontal function and are less likely to experience future cognitive loss^20^. This finding motivates current research on frontal/executive function in the transition from normal cognition to MCI^21^and from MCI to AD^9–11^.

Despite the positive game experience and effective frontal training, the depression score was not significantly changed during the trial period. In selecting an assessment tool, we aimed to use a specialized scale for the geriatric population while imposing a minimal burden on participants. Accordingly, we chose the GDS due to its widespread use in clinical settings, familiarity among researchers, and its ease of administration to patients^22^. However, compared to more comprehensive scales such as the Hamilton Depression Rating Scale (HAM-D), Beck Depression Inventory (BDI), and the Center for Epidemiologic Studies Depression Scale (CES-D), the GDS has limitations in thoroughly evaluating various aspects of depression—including mood, guilt, suicidality, insomnia, anxiety, weight loss, and somatic symptoms^23^. Moreover, the GDS is less sensitive than those scales in tracking longitudinal changes^23^. Additionally, unlike the Cornell Scale for Depression in Dementia (CSDD), it does not incorporate information provided by caregivers^24^. As this study was an exploratory clinical trial, future confirmatory trials should collect data using scales that encompass more multidimensional aspects of depression.

From a different perspective, many participants in this study stated that although the tasks in our intervention were enjoyable to perform, they occasionally became frustrated with the game’s task outcomes. Self-efficacy is defined as an affirmation of ability and strength of belief, whereas self-confidence solely refers to the degree of certainty in outcome^25^. Regarding the relationship between depression with self-confidence and self-efficacy, increasing both components would be beneficial for preventing depression in MCI. Future research should be done to replicate this result using a control group and a larger sample size.

The small sample size without a control group and the relatively short test–retest interval limits the generalizability of the results. However, participants with MCI demonstrated significant cognitive improvement and strong engagement with the intervention. The goal of this study was to determine the usability and feasibility of video game-based digital therapeutics, and this outcome brings to mind the oxymoron “serio ludere”. The most positive conclusion, perhaps, is that older adults with MCI find narrative in video games to be relatable and are driven to explore the game not only for training purposes but also to solve a mystery and look for more stories. As a flâneur exploring the game, our senior users might come to understand that error with flow is merely a fresh tool for discovering the truth. Naturally, going through a nonlinear experience might be painful and frustrating, but never let those despair^26^.

## Methods

### Study design and participants

The study was conducted between May 2023 and August 2023. All participants recruited from the Department of Neurology at Asan Medical Center were ≥ 40 years old and diagnosed with MCI according to Petersen’s criteria^27^. Patients who had any of the following were excluded: dementia due to AD, vascular dementia, or Parkinson’s disease; cognitive decline secondary to other causes, such as folate or vitamin B12 deficiency, syphilis, brain tumors, seizures, encephalitis, and delirium; less than a high school education; neurological and psychiatric conditions that make it difficult to perform tablet-based cognitive tasks, such as dominant hand paralysis or neglect syndrome, or language or visual impairment. All participants enrolled in the study provided written informed consent.

Among 20 eligible patients, 2 participants dropped out during the session and 18 individuals completed the intervention. One outlier exceeded 3SD from their SNSB-II Color Word Stroop Test (CWST) color reading z-score because of color blindness and was removed from the data analysis according to the Standard Deviation Method^28^. Finally, 17 individuals were included in this study.

### Ethics declarations

This study was approved by the Institutional Review Board at Asan Medical Center (IRB No. S2023-0114–0001). Written informed consent was obtained from all eligible patients or their legally authorized representatives. All methods were performed in accordance with the relevant guidelines and regulations of the Asan Medical Center Ethics Committee and the Declaration of Helsinki. All collected data were anonymized to prevent personal identification and then used for analysis. Clinical data, cognitive training data, and survey responses were stored in encrypted files, with access restricted to authorized researchers only.

### Cognitive intervention

**Fig. 3:**
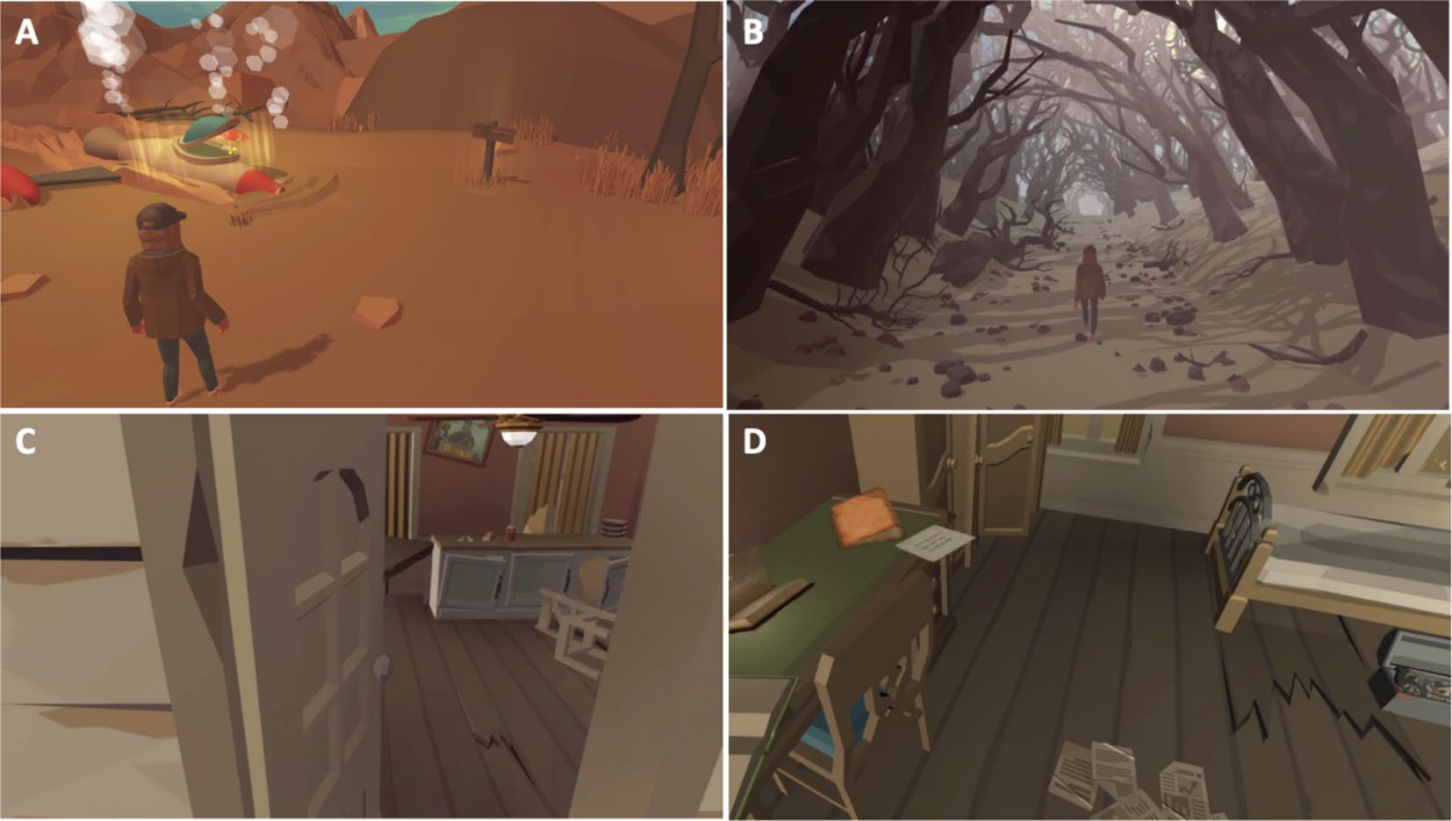
In-game display of the intervention. Participants should control the character to interact with various objects in game through the touch interface. Objects that the user should touch are gleaming and are related to the narrative of the game intervention. Key scene snapshots from the game narrative: Crash Landing (**A**), Into the Forest (**B**), Unlocking the Sealed House (**C**), Discovery of the Compass and Map (**D**).

**Fig. 4:**
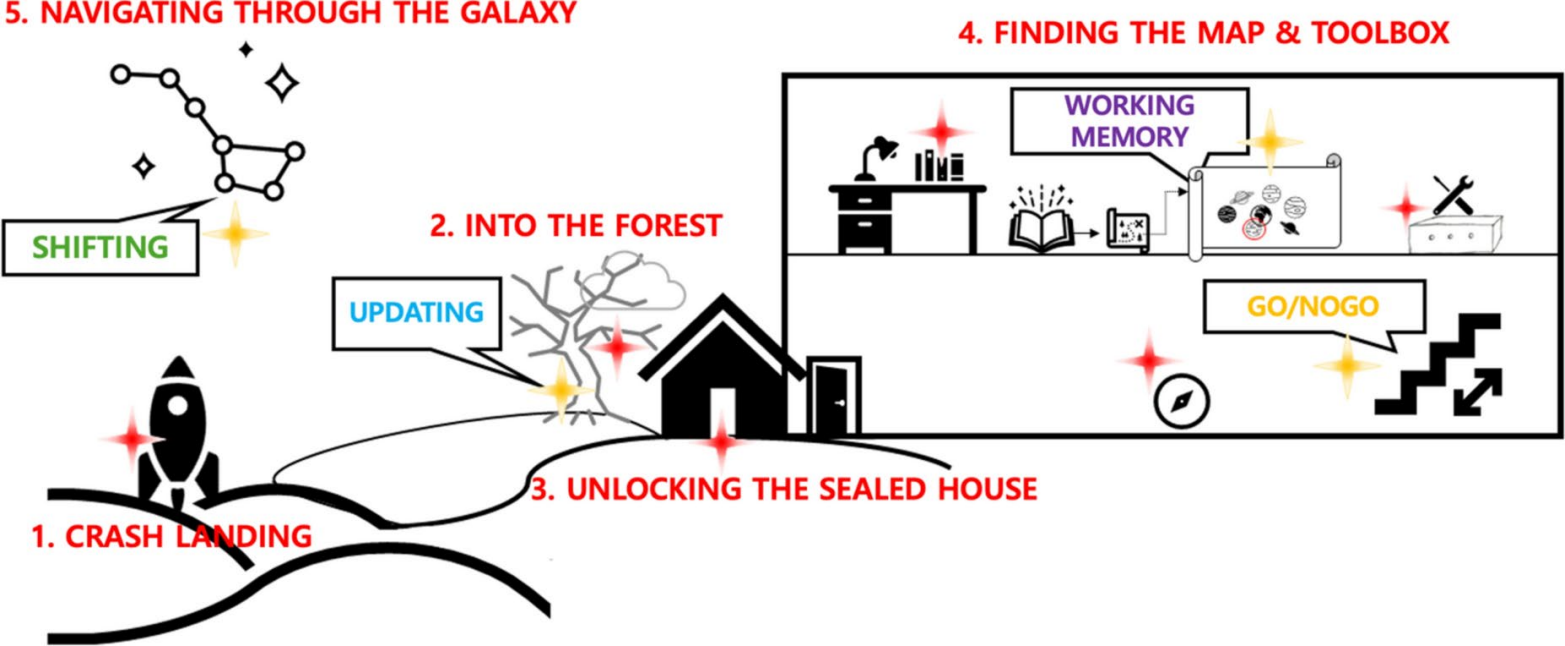
Storyline with tasks of the intervention. The red text in the figure corresponds to the sequence of the game’s storyline, following the numbered order. Each box contains the tasks being trained at the corresponding points in the game. (1) The protagonist crash-lands on an unknown planet, waking up in a deserted desert with no memory. (2) After discovering a locked house and a large tree nearby, he finds a sarcophagus with mysterious symbols and a notebook. Decoding the symbols using the notebook (n-back task), they open the sarcophagus and find a key. (3) Returning to the house, they unlock the door and explore, finding a compass, notes, and a toolbox (Go-No Go task). (4) Upstairs, they discover books, a brochure for another planet, and a map (Corsi block-tapping task). (5) Using the toolbox, the protagonist repairs the plane and, with the help of the compass, sets a course for a planet with people (Trail-making task). All in-game quests woven into the storytelling of the game are related to four frontal functions as shown in the figure above.

The cognitive intervention was designed as a third-person, tablet-based mystery adventure game incorporating cognitive training mechanisms (Fig. 3**, Supplementary Table 1**). This self-guided at-home intervention consisted of four frontal tasks designed to train shifting/divided attention, inhibition, updating, and working memory. All tasks were given with tutorials, woven into the narrative of the game in the form of in-game quests (Fig. 4), with a leveling system adjusted to each patient’s performance.

Participants completed four frontal cognitive tasks on study-provided Galaxy S8 tablets per day. The daily session of cognitive intervention takes approximately 20–30 min, and should be done at least 5 days per week, for 4 weeks (± 1 week). The study coordinator monitored the adherence of the participants using a web dashboard created by Nunaps Inc. After the completion of 4-week (± 1-week) sessions, the participants visited a center for follow-up assessments.

The video game-based cognitive intervention used in this study was developed by four video game developers who are co-authors of this study. H Choi and E Seong implemented the game mechanics, including the narrative design and the creation of specific mini-games. YH Ryu contributed to the graphic design and optimized the user interface. I Park, as the development team leader, guided and managed the overall game development process.

### Outcomes

Frontal function was estimated from the z-scores of five frontal/executive function subtests from the Seoul Neuropsychological Screening Battery, 2^nd^Edition (SNSB-II)^29^: CWST for inhibitory control^30^, COWAT for word fluency^31^, DSC for processing speed^32^, TMT-E Part A for attention^33^, and Part B for divided attention and shifting. Spatial memory span range of 2–9 from the Corsi block-tapping test was also chosen to evaluate the effectiveness of working memory and attention, which are considered important executive functions^34^. In addition, to assess changes in depressive symptoms before and after the intervention, we evaluated participants using the GDS^35^, which ranges from 0 to 30 points. A score of 18 or higher on this scale is indicative of significant depression. Lastly, a user experience interview was conducted during the follow-up evaluation to assess game-experience and feasibility of the intervention for MCI (**Supplementary Table 2**). The individual most familiar with the patient’s condition and could observe the intervention process at home was registered as the primary caregiver for the study. While they did not participate directly in the intervention, their role was to monitor the participant’s engagement in cognitive training and to observe cognitive and emotional changes in daily life. This assessment was conducted through an interview with the primary caregivers at the end of the intervention, providing qualitative information. The goal was to explore changes in daily life, the patient’s attitude toward treatment, and emotional responses—factors that may be difficult to capture through quantitative measures like frontal function tests and the GDS. All assessments except for the user experience interview were performed at baseline and at 4-week (± 1-week) follow-up.

### Statistical analysis

The frontal test score was estimated by averaging the z-scores from CWST (Color reading), COWAT (Phonemic fluency), DSC, and TMT Part A and B^36^. These z-scores from each subtest were standardized raw scores based on the published normative data with matched age and education level^29^. Because the data for normality did not meet the assumptions of the test, the Wilcoxon signed rank test was performed to assess the changes in the mean z-scores from SNSB-II frontal/executive tests, the block span from Corsi block-tapping test, and the GDS score from pre- to post-intervention. Multiple comparison correction was performed using Bonferroni methods. Data management and analysis were performed using R software (version 4.3.1), and P-values < 0.05 were considered significant.

## Supplementary Information

Supplementary Information.

## Data Availability

The datasets generated or analyzed during the current study are not publicly available due to privacy policy of the patients in the Asan Medical Center but are available from the corresponding author only on reasonable request.
