# Supplementary material for: Narrative mobile video game-based cognitive training to enhance frontal function in patients with mild cognitive impairment
Source: Sci Rep. 2025 Jan 2;15:195. doi: 10.1038/s41598-024-84086-9 (PMC11696466; doi:10.1038/s41598-024-84086-9)
Supplement: Supplementary file 1 — Supplementary Information. [file 41598_2024_84086_MOESM1_ESM.docx]

**Supplementary Material**

**Supplementary table 1.** Frontal tasks of the intervention.

| **Tasks** | **Targeted domain** | **Description of the tasks** |
| --- | --- | --- |
| Connecting the 12 zodiac signs | Mental shifting ability | User should follow the zodiac signs in order. |
| Climbing stairs | Inhibition | When climbing the stairs, user should discriminate each stair to step on or not. |
| Password hidden in calendar | Updating | Concentrating on calendar turning, user should indicate whether the stimulus on calendar corresponds to the stimulus of earlier page. |
| Find out path of planets | Visuospatial working memory | User needs to tap the sequence of the presented stimuli. |

**Supplementary table 2.** Semi-structured questionnaire for user experience interview

| Question Items |
| --- |
| (1) Have you previously played any other mobile games, or do you currently enjoy any specific games?  (2) How did you find the difficulty level of each task in the game? Which task was the most challenging, and which was the easiest? If you found any tasks difficult, what do you think contributed to that difficulty?  (3) Did you find the game enjoyable? If so, what aspects of the game did you find engaging or fun?  (4) How did you feel about the music in the game? Was it pleasant to listen to?  (5) How did you feel after playing the game?  (6) Did you notice any positive changes in your cognitive abilities after playing the game?  (7) If we further develop this game and conduct a confirmatory clinical trial, would you be interested in participating?  (8) If the game becomes commercialized and available as a product, would you continue playing it?  (9) Would you recommend this game to other MCI patients, or to friends or family members concerned about their cognitive function? |
